# Useful resources: instruments and consumables

**Published:** 2011-12

## IAPB Standard List

The **IAPB Standard List** provides information for eye health providers on a carefully evaluated range of eye care technologies, supplies, and training resources suitable for use in settings with limited resources.

You can now register to use the new interactive, online IAPB standard list **(http://IAPB.standardlist.org)** which allows you to create a ‘basket’ of items that you can print out and use when drawing up an order.

Individuals working for IAPB member organisations, or working in partnership with these organisations, will have access to the full functionality of the site, which includes being able to see the prices of all items and, in the near future, to order directly from suppliers.

Registration may take one or more days to be approved.

Paper copies of the IAPB Standard List can be ordered from Teaching Aids at Low Cost (TALC).

## Talc (Teaching Aids at Low Cost)

PO Box 49, St Albans, Hertfordshire, AL1 5TX, UK. Tel +44 (0) 1727 853869. Email info@talcuk.org

## Teaching and learning resource

**Prozesky D, Stevens S, Hubley J. Effective Teaching and Learning for Eye Health Workers**, International Centre for Eye Health, 2006. Free download (720 KB) on www.cehjournal.org/icehpubs.html or order free of charge from TALC. High-income countries pay £5.

## Videos

**Karen Watts.** Handling and passing of surgical instruments; Instrument care during decontamination and sterilisation; Handling of sterile supplies (all part of the ‘Nursing’ package). Cost: Rs 500 per package + free postage (India only) or US $40 per package for international orders. Order from LV Prasad Eye Institute. Write to Shobha Mocherla, A-V Producer, Central Audio-Visual Unit (CAVU) LV Prasad Eye Institute, LV Prasad Marg, Hyderabad 500 034, India. Email: video@lvpei.org

## Procurement agencies in Africa

**Joint Medical Stores, Uganda.** Plot 1828 Gongonya Rd, PO Box 4501 Kampala. Tel +256 (0)414510096 or toll free (Uganda only): 0800 123124. Email store@jms.co.ug or visit www.jms.co.ug

**Missions for Essential Drugs, Kenya.** PO Box 78040-00507, Viwandani, Nairobi Tel +254 (0)203920102. Email customerservice@meds.or.ke or visit www.meds.or.ke

**Action Medeor, Tanzania.** PO Box 72305, Dar es Salaam, Tanzania. Tel +255 (0)222863136.

**Cameroon Baptist Convention Health Services, Cameroon.**

Cameroon Baptist Convention Health Board, PO Box 1 Bamenda, North West Province, Cameroon. Tel: +237 (0)77964683.

Email: info@cbchealthservices.org or visit www.cbchealthservices.org

## Pre-shipment inspection companies

### INTERTEK

http://www.intertek.com/government/pre-shipment/inspection/

### COTECNA

http://www.cotecna.com/COM/EN/psi.aspx

### SGS

http://www.sgs.com/pre_shipment_inspection__import_verification.htm?serviceId=6942&lobId=5549

## Courses: care and maintenance of ophthalmic instruments

**LAICO, Aravind Eye Care Systems (AECS)** runs six-week training courses in Madurai for instrument technicians. These are repeated four times a year (US $325). LAICO offers shorter courses on invitation at a range of different training centres. The LAICO team has also helped to establish instrument maintenance training centres at the National Eye Institute, Kaduna, Nigeria; the National Institute of Ophthalmology, Hanoi, Vietnam, and Kikuyu Eye Unit, Kenya. Write to Prof V Srinivasan, LAICO, 72, Kuruvikaran Salai, Gandhi Nagar, Madurai 625 020, Tamil Nadu, India. Email: v.srinivasan@aravind.org or visit www.aravind.org/education/coursedetails.asp

